# Customized eye modeling for optical quality assessment in myopic femto-LASIK surgery

**DOI:** 10.1038/s41598-021-95730-z

**Published:** 2021-08-06

**Authors:** Gongpu Lan, Jun Zeng, Wenjie Li, Guoqin Ma, Qun Shi, Yue Shi, Yicheng Wang, Jingjiang Xu, Yanping Huang, Jia Qin, Jinping Feng, Haishu Tan, Lin An, Xunbin Wei

**Affiliations:** 1 School of Physics and Optoelectronic Engineering, Foshan University, Foshan, 528000 Guangdong China; 2 Innovation and Entrepreneurship Team of Guangdong Pearl River Talents Program, Guangdong Weiren Meditech Co., Ltd, Foshan, 528000 Guangdong China; 3 Guangdong-Hong Kong-Macao Intelligent Micro-Nano Optoelectronic Technology Joint Laboratory, Foshan University, Foshan, 528000 Guangdong China; 4 The First People’s Hospital of Foshan, Foshan, 528000 Guangdong China; 5 School of Mechatronic Engineering and Automation, Foshan University, Foshan, 528000 Guangdong China; 6 Institute of Engineering and Technology, Hubei University of Science and Technology, Xianning, 437100 Hubei China; 7 Biomedical Engineering Department, Peking University, Beijing, 100081 China; 8 Key Laboratory of Carcinogenesis and Translational Research, Peking University Cancer Hospital & Institute, Beijing, 100142 China; 9 State Key Laboratory of Oncogenes and Related Genes, Shanghai Cancer Institute, Med-X Research Institute and School of Biomedical Engineering, Shanghai Jiao Tong University, Shanghai, 200030 China

**Keywords:** Refractive errors, Applied optics

## Abstract

Refractive surgery is recognized as an effective method for myopia treatment, but it can induce night vision disturbances such as glare. We present an eye modeling method for the optical quality assessment in response to the structural changes in the eyes by femto-LASIK surgery. Customized eye models were built from the measurements of 134 right eyes pre- and post-operatively. Optical performance was evaluated using spot diagrams, point spread functions (PSFs), modulation transfer functions (MTFs), and chromatic aberrations at various fields (0°–30°), different pupil diameters (2–6 mm), and initial myopias (− 1.25 to − 10.5 D). Pupil size and initial myopia are the two major factors that affect visual performance of post-operative eyes. The results of spot diagrams, PSFs, and MTFs indicated that post-operative visual performance deteriorated as the visual field and pupil size increased, and it was significantly influenced by initial myopia. Post-operative chromatic aberrations were also affected by initial myopia. As pupil size increased, the post-operative longitudinal chromatic aberrations tended to decrease slightly, while the transverse chromatic aberrations remained similar. The use of eye modeling for refractive surgery assessment could possibly provide a more personalized surgical approach, could improve the prediction accuracy of refractive surgery outcomes, and promote the invention and development of better surgical methods.

## Introduction

Myopia (also called short-sightedness or near-sightedness) is the most common form of refractive error and has emerged as a major public health issue globally^1^. In urban areas of east and southeast Asia, approximately 80–90% of children in high school are myopic, and 10–20% have high myopia^2^. By the year 2050, half of the world’s population is predicted to have myopia, and approximately one billion people are predicted to have high myopia^3^. Since the human cornea is the most accessible part of the eye with two-thirds of the total ocular refractive power, refractive surgery on the cornea has become the mainstay of myopic correction^4^.

Laser-assisted in situ keratomileusis (LASIK)^5^ is a popular type of corneal refractive surgery for myopia treatment. During the LASIK procedure, a thin-hinged corneal flap is created and folded back, and the exposed stroma is removed using an excimer laser so that the central corneal is flattened to compensate for the longer axial length or the excessive refractive power of the myopic eye. With the introduction of a femtosecond laser for corneal flap creation, femto-LASIK refractive surgery has demonstrated improved cutting results with minimal distorted flap edge^6^, better prediction of flap thickness^7^, reduced intra-operative, flap-related complications^8^, and better biomechanical stability^9^. However, night vision disturbances, such as glare, halos, and starbursts, are often reported by patients and in fact are the most significant complaints after laser refractive surgeries^10–13^. The cause of night vision disturbances is mainly considered to be that the surgery can induce high-order aberrations when the magnitudes of the low-order sphero-cylindrical aberrations are reduced. When the pupil is enlarged to adapt to a low light illumination condition, the marginal rays of vision at the transition zone of surgery tend to be diverged, so visual aberrations are produced and vision quality is deteriorated^14^. Therefore, understanding the changes in visual performance caused by refractive surgery is important to reduce the night vision disturbances and to improve patient satisfaction^15,16^.

Zernike polynomials have been used as standards to represent the low- and high-order monochromatic wavefront aberrations of the eyes^17^. It has been commonly assumed that the vision quality decreases as the root mean square (RMS) values of the high-order ocular wavefront aberrations increase, and wavefront-guided refractive surgery solutions have been developed to reduce the residual RMS values of high-order aberrations in myopic eyes^18–21^. However, the decrease or increase in the RMS values of the Zernike wavefront aberrations cannot be simply linked to the decrease or increase in the visual performance^22,23^. It has been demonstrated that one term of Zernike aberration can compensate another and improve the visual quality (e.g., the modulation transfer function, MTF) and image quality^24^. In addition, specific combinations of Zernike modes can interact to improve acuity despite an increase of the total RMS wavefront errors^22,23^. The RMS values of ocular aberrations are too rough and simple to judge optical performance, whereas the analysis of all the Zernike terms is too complicated to make the assessment. In addition, with the Zernike analysis method it is hard to describe any field-dependent aberrations or chromatic aberrations.

The eye modeling method has been used as a computational tool in visual science since the mid-nineteenth century to establish a conceptual framework for explaining optical phenomena in vision, to predict how the changes in ocular biometry affect refraction and aberrations, to explore the limitations imposed on vision by the optical system of the eye, or to provide a physical standard used to design or test the ocular imaging instruments^25^. To meet each of the aforementioned purposes, a variety of optical models of the human eye have been developed with different levels of complexity^25^—ranging from single-surface models (reduced eyes)^26^ to sophisticated finite-element models that describe layered features of each component^27,28^, from models with all spherical surfaces to aspherical^29^ or even high-order Zernike surfaces^30,31^, from a symmetrical structure^27^ to asymmetrical structures with tilted and decentered ocular components^32^, from models with constant refractive index elements to models with gradient refractive index^33^ or continuously accommodable lenses^31,34^. The use of a suitable eye model is mainly based on the principle whereby this eye model can provide valid prediction results for a particular purpose with enough simplicity, but does not necessarily rely on whether the model is anatomically or mechanistically right^25,29^. The goal of the present study was to develop an analytical eye model for accurately evaluating the optical quality change in response to the structural changes in patients’ eyes by femto-LASIK surgery. To balance the functional accuracy and anatomical simplicity, we prioritized the modeling accuracy of the anterior segment topography (especially cornea) using sixth-order Zernike polynomials, while we simplified other parts of the eye (i.e., the lens, vitreous body, and retina) as symmetrical components with aspherical surfaces and constant refractive index values. Ocular geometries were clinically measured and calculated before and after the surgery from 134 right eyes, and the customized eye models were built in Zemax (Zemax, LLC). We performed the optical quality analysis for the whole eye, including the spot diagram, point spread function (PSF), MTF, and chromatic aberrations. We evaluated the imaging quality at various pupil diameters (2–6 mm), different fields of view (0°–30°), and different initial myopic diopters (− 1.25 to − 10.5 D). The use of customized eye models in the prediction of refractive surgery can relate the effect of anterior segment change (especially corneal shape change) on the retinal imaging quality performance more directly using the standard optical metrics (e.g., PSF and MTF). We aimed to provide a reliable evaluation tool with the potential to aid in the development of better surgery methods and strategies to reduce surgery-induced eye symptoms and increase post-operative visual performance.

## Methods

### Patient population

Data were collected from 134 right eyes of 134 patients (72 females and 62 males; ages: 28 ± 6 years; spherical errors: − 1.25 to − 10.5 diopters [D]; astigmatisms: <  − 3.0 D). No patient had ocular disease (except myopia), previous ocular surgery, or any systemic condition that could have affected this study. The research protocol was approved by the Institutional Review Board of Foshan University and adhered to the tenets of the Declaration of Helsinki. The surgery was performed in the First People's Hospital of Foshan, from March 2018 to July 2020. The study was enrolled after the approval of the Ethics Committee of the hospital and signed informed consent was collected from all participants.

### Femto-LASIK surgery

Preoperative ophthalmic examinations were performed to exclude participants with systemic disease, ocular diseases other than refractive errors, abnormal corneal topographies, or thin corneas (≤ 450 μm). Patients were also examined postoperatively at 1 day, 1 week, 1 month, 3 months, 6 months, and 1 year for follow-up assessments. These ocular examinations included the best corrected vision acuity, subjective and objective refraction, anterior segment geometry (Pentacam, Oculus Optikgeräte GmbH, Wetzlar, Germany), axial dimensions of ocular structures (Lenstar LS900 biometer, Haag-Streit, USA), slit-lamp exam, fundus examination, and intraocular pressure measurement.

Levofloxacin eye drops were used three days before the surgery. In femto-LASIK, an 8.5-mm corneal flap was cut at a 100-μm depth by an IntraLase FS 60 Femtosecond laser (IntraLase, Irvine, California), and the stroma was removed using an EX500 (Alcon) excimer laser that was guided by Q-algorithm^35^. The optical region was 6.5 mm, the residual corneal stroma thickness was no less than 300 µm, and the postoperative corneal thickness was no less than 400 µm. The flap was carefully repositioned after laser treatment. Patients were required to wear bandage contact lenses (Bausch) to avoid corneal flap displacement and to promote epithelial repair. The contact lenses were removed after 1 day, and the postoperative medication was started. Tobramycin dexamethasone eye drops were used 4 times per day for 1 week, levofloxacin eye drops were used 4 times per day for 1 to 2 weeks, and sodium hyaluronate eye drops were used 4 times per day for 2 to 3 months.

### Eye modeling

**Figure 1:**
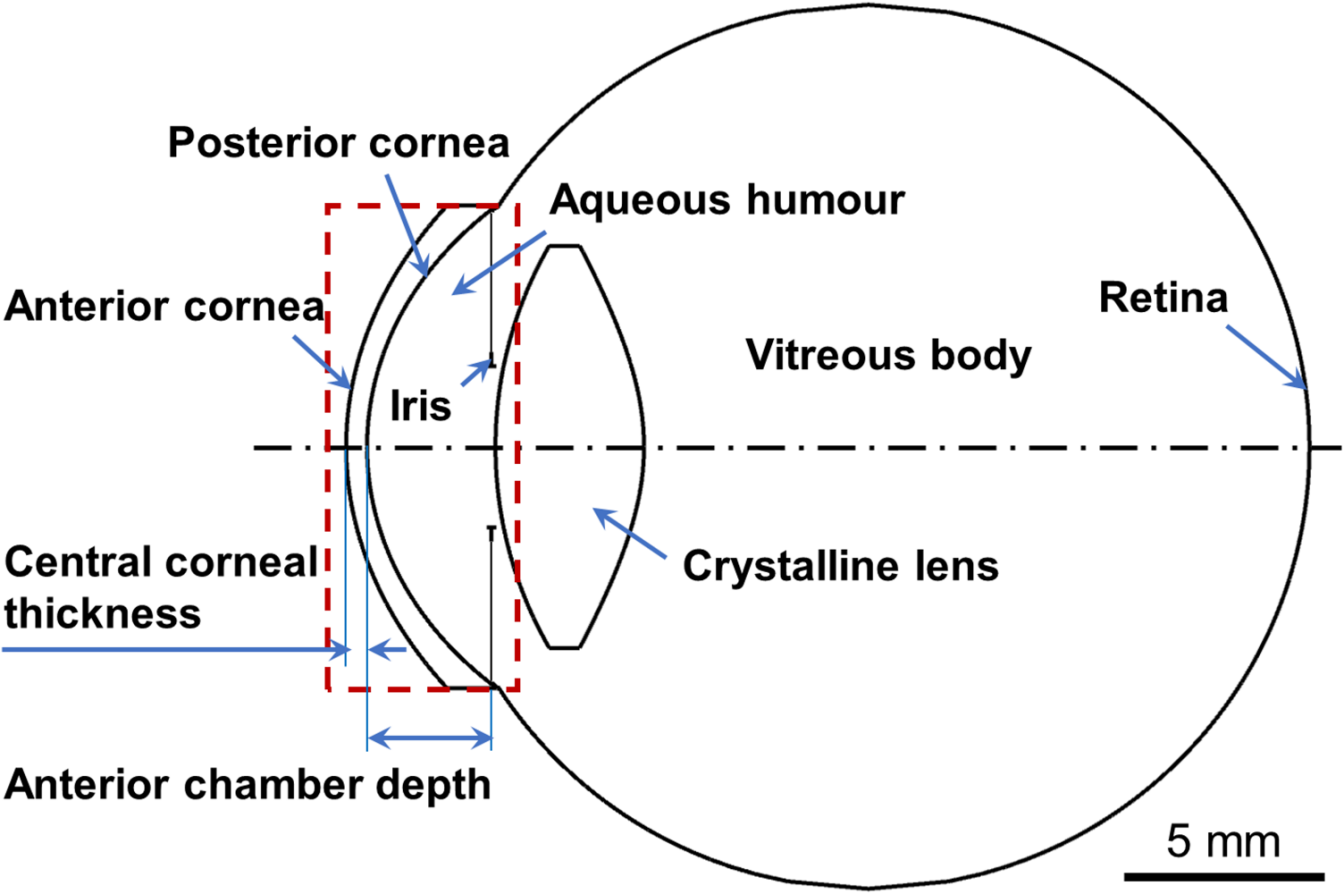
Schematic of a customized human eye model to access the ocular structure change in response to femto-LASIK. The customized eye model was started from a schematic eye model^29^ and then was built by replacement of the measured or estimated ocular biometric parameters.

The customized eye model of each patient’s right eye was built using Zemax OpticsStudio (Zemax, LLC), as shown in Fig. 1. We assumed that the refractive indexes and Abbe-numbers relative to the green light (wavelength: 588 nm) were 1.377 and 56.28 for cornea, 1.337 and 52.659 for aqueous humor, 1.42 and 51.226 for crystalline lens, and 1.336 and 53.342 for vitreous body^29^. The anterior segment was built from the measurement of Pentacam using a rotating Scheimpflug camera with 138,000 points in 2 seconds^36^. The measurement parameters included the radii of curvatures and asphericity values of anterior and posterior corneas, central corneal thickness, and the anterior chamber depth. Particularly, the heights of the anterior and posterior corneas were fitted to a serious of Zernike polynomials in a 6-mm diameter according to the OSA standard^17^:

1 $$ z = \frac{{r^{2} /R}}{{1 + \sqrt {1 - (1 + Q)r^{2} /R^{2} } }} + \sum\limits_{i = 0}^{n} {\sum\limits_{j = 0}^{m} {\alpha_{i}^{j} Z_{i}^{j} (\rho ,\phi ),} } $$

where *z* is the sag of cornea surface; *R* is the average corneal radius; *r* is a radial ray coordinate; and *Q* is a conic constant (asphericity). The conic constant *Q* is less than − 1 for hyperbolas, − 1 for parabolas, between − 1 and 0 for ellipses, 0 for spheres, and greater than 0 for oblate ellipsoids. Zernike polynomials are defined with polar coordinates (*ρ*, *φ*), where *ρ* is a radial coordinate ranging from 0 to 1, and *φ* is an azimuthal component ranging from 0 to 2π. Z_*i*_^*j*^ represents a Zernike polynomial, and *α*_*i*_^*j*^ is the coefficient for Z_*i*_^*j*^, where *i* represents a radial order (the degree of the polynomial), and *j* represents a meridional frequency (the number of cycles of sinusoidal variation across 360° of meridian). The value of *i* (*i* = 0 to *n*) is a positive integer or zero, and the value of *j* (*j* = 0 to *m*) can only be − *i*, − *i* + 2, − *i* + 4, … *i*. The negative value of *j* represents that the meridional component is in a sine phase, and the positive value of *j* indicates a cosine phase with respect to the horizontal direction. Each Zernike polynomial Z_*i*_^*j*^ can be represented as^17^

2 $$ Z_{i}^{j} (\rho ,\theta ) = \left\{ {\begin{array}{*{20}c} {\sqrt {\frac{2(i + 1)}{{1 + \delta_{j0} }}} \sum\limits_{s = 0}^{(i - \left| j \right|)/2} {\frac{{( - 1)^{s} (i - s)!}}{s![0.5(i + \left| j \right| - s]![0.5(i - \left| j \right| - s]!}\rho^{i - 2s} } \cos j\theta ;for^{{}} j \ge 0} \\ { - \sqrt {\frac{2(i + 1)}{{1 + \delta_{j0} }}} \sum\limits_{s = 0}^{(i - \left| j \right|)/2} {\frac{{( - 1)^{s} (i - s)!}}{s![0.5(i + \left| j \right| - s]![0.5(i - \left| j \right| - s]!}\rho^{i - 2s} } \sin j\theta ;for^{{}} j < 0} \\ \end{array} } \right\}, $$

where *δ*_*j0*_ is the Kronecker delta function, *δ*_*j0*_ = 1 for *j* = 0, and *δ*_*j0*_ = 0 for *j* ≠ 0.

Currently, clinical measurements of lens biometric parameters—such as radii of curvature, asphericities, and refractive index distribution—are challenging to obtain^37^. Previous studies have shown very weak relationships between the lens biometric parameters and refraction, such as *P*-value = 0.867 between the lens thickness and refraction, and *P*-value = 0.848 between lens power and refraction^25^. In this study, we assumed that the lens biometric parameters were unaffected by refraction, and we adapted previous estimation methods^25,38–42^ to build the parameters of the crystalline lens in each eye model. We set the tilt and decentration of the lens as zero to simplify the estimation of the lens parameters. The equivalent lens power *P*_*L*_ can be calculated based on the measured parameters using Bennett’s method^39^:

3 $$ P_{L} = \frac{{ - 1000n_{h} (S + P_{C} )}}{{1000n_{h} - (T_{AC} + C_{1} T_{L} )(S + P_{C} )}} + \frac{{1000n_{h} }}{{ - C_{2} T_{L} + T_{V} }}, $$

where *S* is the spherical refraction of the eye (unit: D); *P*_*C*_ is the power of the cornea (unit: D); *T*_*AC*_, *T*_*L*_, and *T*_*V*_ are the thickness/depth of the anterior chamber, lens, and vitreous body, respectively (unit: mm); *n*_*h*_ = 1.337 is the refractive index of the aqueous humor; *c*_*1*_*T*_*L*_ is the distance between the anterior lens surface and the first lenticular principal plane, and *c*_*2*_*T*_*L*_ is the distance between the posterior lens surface and the second lenticular principal plane, where the constants are *c*_*1*_ = 0.571 and *c*_*2*_ =  − 0.378^39,40^. We used the *T*_*AC*_ values from Pentacam measurements and the *T*_*L*_ and *T*_*V*_ values from Lenstar measurements. We estimated the radii of curvature of the arterial lens surface (*R*_*La*_) and the posterior lens surface (*R*_*Lp*_) using a linear regression fitting result provided by Rozema et al.^41^:

4 $$ \left\{ \begin{gathered} R_{La} = 26.02 - 2.7T_{L} - 0.2P_{L} , \hfill \\ R_{LP} = - 16.675 + 1.696T_{L} + 0.126P_{L} . \hfill \\ \end{gathered} \right. $$

The values of asphericities (conics, or *Q*-values) of the unaccommodating lens vary over a wide range in different eye models and literature due to the difficulties of measurement^25,27,29,34,37,42–44^. Here we estimated the asphericities of the arterial and posterior lens surface using the fixed values of − 3.13 and − 1, respectively, based on the Navarro eye model^34^.

The retinal shapes of emmetropic and myopic eyes were described by Atchison et al. using a non-rotationally symmetrical ellipsoid fitting method^45^. They found the shapes of retinas were oblate in most of emmetropic eyes. As myopic diopters increased, the ellipsoid dimensions increased, with the axial dimension increasing more than the vertical dimension, which in turn increased more than the horizontal dimension^45^. The mean values of the vertex radii of curvature (*R*_*R*_) and asphericities (*Q*_*R*_) of retina are described as^45^

5 $$ \left\{ \begin{gathered} R_{R} = - 12.815 - 0.045S, \hfill \\ Q_{R} = 0.26 + 0.022S. \hfill \\ \end{gathered} \right. $$

In all cases, an object was placed at infinity so that the entered lights had plane wavefronts without any aberrations, and the crystalline lenses were unaccommodated. The object was ideally focused on the retinas at the visual field of 0° for all eyes. For the pre-operative eye models, ideal spectacles were used to achieve the optimized visual acuity since most myopic subjects wear spectacle lenses in their daily lives to compensate for the refraction errors. For the post-operative eye models, the retina positions were shifted to focus the light beams. Zemax simulations were performed on the pre- and post-surgical eyes to compare their optical performances, and these included the metrics of the geometry-optics-based spot radius diagram and chromatic aberration, and the fast Fourier transform (FFT)-based polychromatic PSF and polychromatic MTF.

### Statistics

The pre- and post-operative ocular diopters and corneal geometry parameters were compared. Linear regressions were performed to illustrate the surgically induced changes in these parameters in response to the initial myopia. The Pearson correlation coefficient (*r*) and the level of significance (*P* value) were used to investigate the strengths of the correlations. The value of *r* ranges from − 1 to 1, where *r* = 1 indicates a perfect positive correlation, *r* =  − 1 indicates a perfect negative correlation, and *r* = 0 indicates no linear correlation. The statistics were considered significant when *P* < 0.05, and extremely significant when *P* < 0.01. Optical performance was evaluated on eye models based on the initial myopic diopters, which were mild myopia group (0 to − 3 D), medium myopia group (− 3 to − 6 D), and high myopia group (over − 6 D). The optical quality metrics of PSF, MTF, and chromatic aberrations were analyzed at different pupil diameters (2–6 mm) and at different fields of view (0°–30°), and they were compared based on the mean and standard deviation (SD) values for each initial myopia group.

## Results

### Ocular parameters

**Figure 2:**
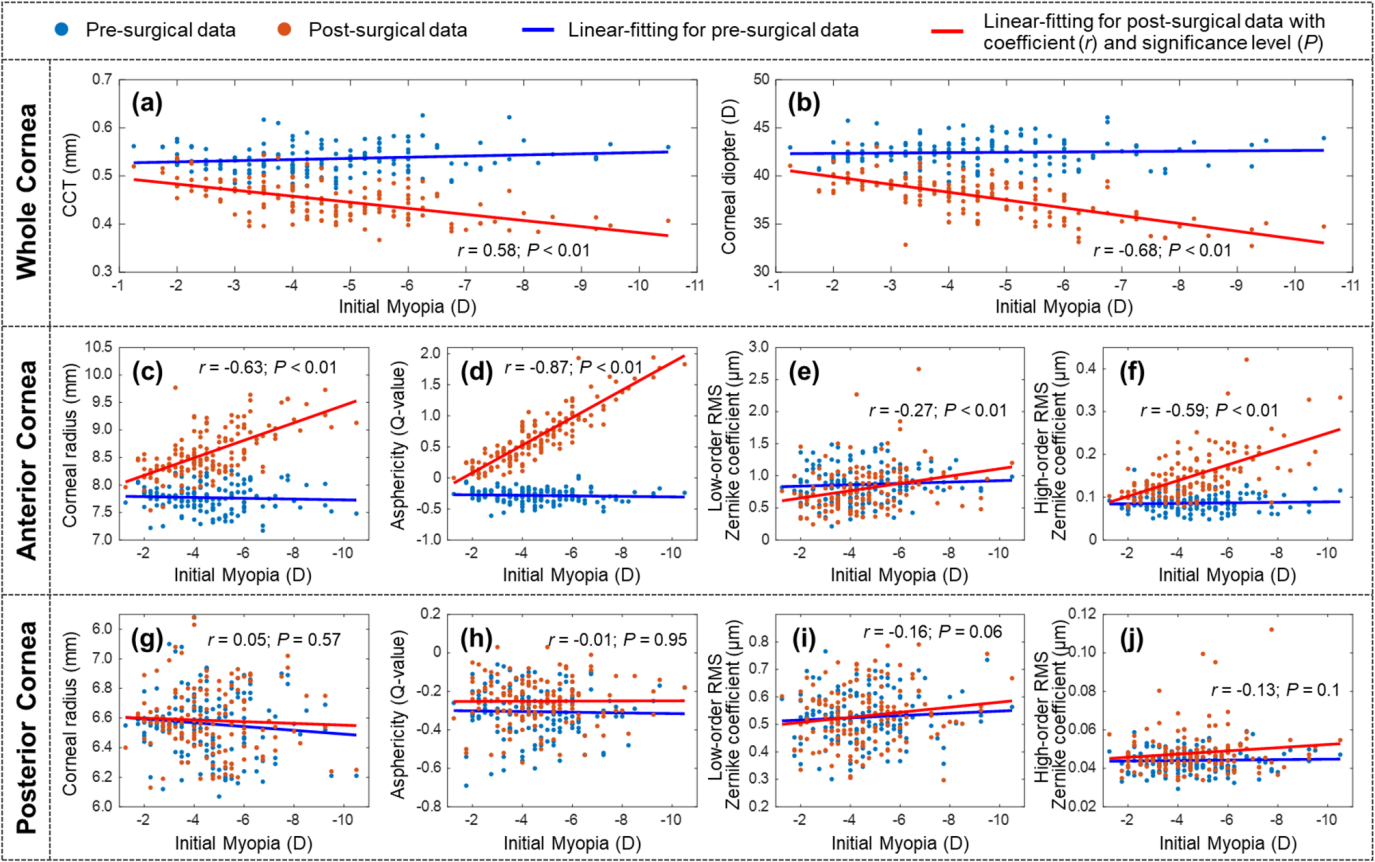
Comparison between the corneal parameters of 134 right eyes before and after the femto-LASIK surgery in response to the initial myopia (− 1.25 to − 10.5 D). These parameters include: (**a**) corneal center thickness (CCT); (**b**) corneal diopter; (**c**) and (**g**) anterior and posterior corneal radii; (**d**) and (**h**) anterior and posterior corneal asphericities (Q-values); (**e**) and (**i**) anterior and posterior corneal low-order (0–2) RMS values in Zernike terms; (**f**) and (**j**) anterior and posterior corneal high-order (3–6) RMS values in Zernike terms.

Each eye model was composed of two Zernike surfaces representing the anterior and posterior corneas, a stop at the pupil plane to represent the iris, two aspherical surfaces representing the crystalline lens, and an aspherical retina, as shown in Fig. 1. The ocular parameters (mean ± SD) from 134 right eyes are shown in Table 1. The change in corneal geometry is the major outcome of the femto-LASIK surgery and has been considered as the major source of the production of high-order aberrations and night vision disturbances^14–16^; while the parameters of the lens, vitreous body, and retina for each patient were assumed to be unchanged after surgery. Corneal parameters before and after the femto-LASIK surgery were compared in response to the initial myopia (Fig. 2). The pre-surgical corneal parameters were not correlated with the initial myopia. The reductions of corneal central thickness (0.54 ± 0.03 mm to 0.45 ± 0.04 mm) and corneal diopter (42.44 ± 1.38 D to 37.84 ± 2.08 D) were directly caused by the surgery [*P*-values < 0.01, Figs 2a,b]. The anterior corneal geometries were greatly altered by the surgery, and were correlated with the intimal myopic diopters with *P*-values smaller than 0.01 [Fig. 2c–f]. Due to the femto-LASIK surgery, the anterior corneas were flatter, while the cornel radii increased from 7.77 ± 0.25 mm to 8.56 ± 0.43. The asphericity (*Q*) values of anterior corneas increased from − 0.28 ± 0.11 to 0.63 ± 0.44, indicating the corneal shapes changed from prolate ellipsoids to oblate ellipsoids. The high-order (3rd to 6th) RMS values of the Zernike coefficients for anterior corneas were enlarged from 0.09 ± 0.02 to 0.15 ± 0.05 μm, and the post-surgical high-order RMS Zernike coefficients were correlated with the initial myopic diopters [*P* < 0.01, Fig. 2f]. The posterior corneal geometries were changed little without any obvious correlations with the initial myopic diopters [*P* > 0.05, Fig. 2g–j].

**Table 1:** General geometry of eye models for 134 right eyes before and 6 months after femto-LASIK.

			Pre-surgery	Post-surgery
Cornea	Anterior Surface^[a],^*	Radius (mm)	7.77 ± 0.25	8.56 ± 0.43
		Asphericity	− 0.28 ± 0.11	0.63 ± 0.44
		Low-order RMS errors (μm)	0.87 ± 0.27	0.80 ± 0.36
		High-order RMS errors (μm)	0.09 ± 0.02	0.15 ± 0.05
	Posterior Surface^[a],^*	Radius (mm)	6.35 ± 0.23	6.38 ± 0.22
		Asphericity	− 0.31 ± 0.12	− 0.26 ± 0.12
		Low-order RMS errors (μm)	0.53 ± 0.09	0.53 ± 0.10
		High-order RMS errors (μm)	0.04 ± 0.01	0.05 ± 0.01
	Thickness (mm)^[a]^		0.54 ± 0.03	0.45 ± 0.04
	Diameter (mm)^[d]^		12	12
Anterior chamber depth (mm)^[a]^			3.39 ± 0.28	3.16 ± 0.27
Pupil size (mm)^[e]^			2, 4, and 6	
Lens	Anterior Surface	Radius (mm)^[c]^	10.83 ± 0.71	
		Asphericity^[d]^	− 3.13	
	Posterior Surface	Radius (mm)^[c]^	− 7.12 ± 0.45	
		Asphericity^[d]^	− 1.00	
	Thickness (mm)^[b]^		3.79 ± 0.42	
	Diameter (mm)^[d]^		10	
Vitreous body	Length (mm)^[b]^		17.59 ± 0.89	
	Diameter (mm)^[d]^		22	
Retina	Radius (mm)^[c]^		− 12.61 ± 0.08	
	Asphericity^[c]^		0.16 ± 0.04	
	Diameter (mm)^[d]^		22	

*The magnitudes of anterior and posterior corneal surfaces were fitted as sixth-order Zernike polynomials.

^[a]^Measured by Pentacam (Oculus Optikgeräte GmbH).

^[b]^Measured by Lenstar LS900 (Haag-Streit).

^[c]^Estimated based on Eqs. (3–5)^39,41,45^.

^[d]^Estimated based on the Navarro eye model^34^.

^[e]^Set values for Zemax simulation.

### Optical performance analysis

**Figure 3:**
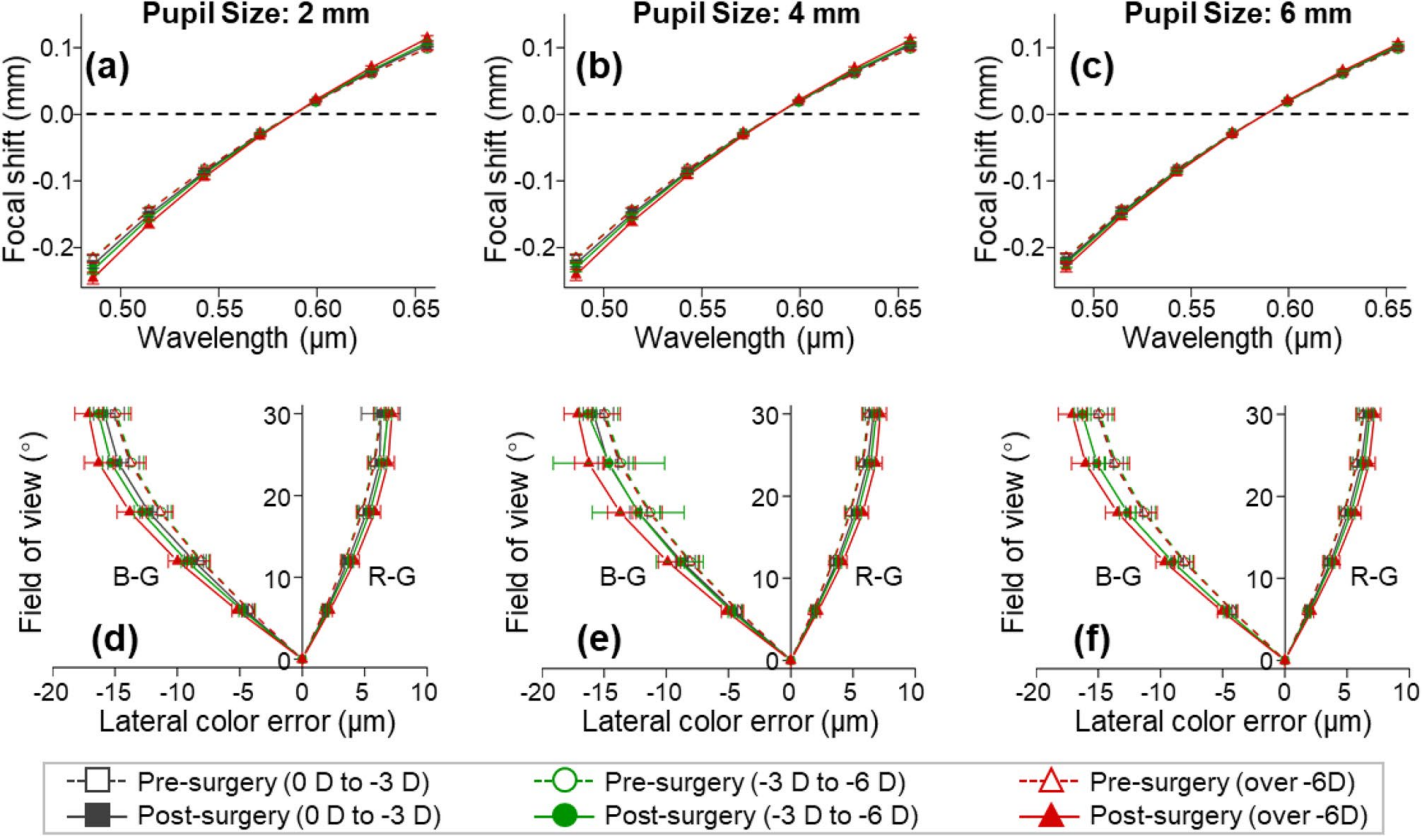
Chromatic aberration analysis (mean ± SD) at retina before and after femto-LASIK. R: red light, 656.273 nm; G: green light, 587.562 nm; B: blue light, 486.133 nm. (**a**–**c**) show the longitudinal chromatic focal shifts relative to green light (587.562 nm) at 2-mm, 4-mm, and 6-mm pupil sizes, respectively. (**d**–**f**) show the transverse chromatic aberrations across the field of view (0°–30°) at 2-mm, 4-mm, and 6-mm pupil sizes, respectively.

Optical performance was evaluated and compared between the preoperative eyes with spectacles and the postoperative eyes without spectacles at various fields (0°–30°), pupil diameters (2–6 mm), and initial myopias (− 1.25 to − 10.5 D). Ocular chromatic aberration is an optical distortion in which the focusing positions, in either the longitudinal (axial) or the transverse (lateral) direction, are wavelength-dependent^46–48^. Figure 3 shows the analysis of the longitudinal chromatic aberrations [Fig. 3a–c] and transverse chromatic aberrations [Fig. 3d–f] in the visible light spectrum (red light: 656.273 nm, green light: 587.562 nm, and blue light: 486.133 nm). Before the femto-LASIK surgery, neither the longitudinal nor the transverse chromatic aberrations were dependent on pupil size or initial myopia. Both aberrations increased slightly after the surgery, and increased as the initial myopic diopter increased. As the pupil size increased, the longitudinal chromatic aberrations after refractive surgery tended to decrease slightly, while the transverse chromatic aberrations remained similar.

**Figure 4:**
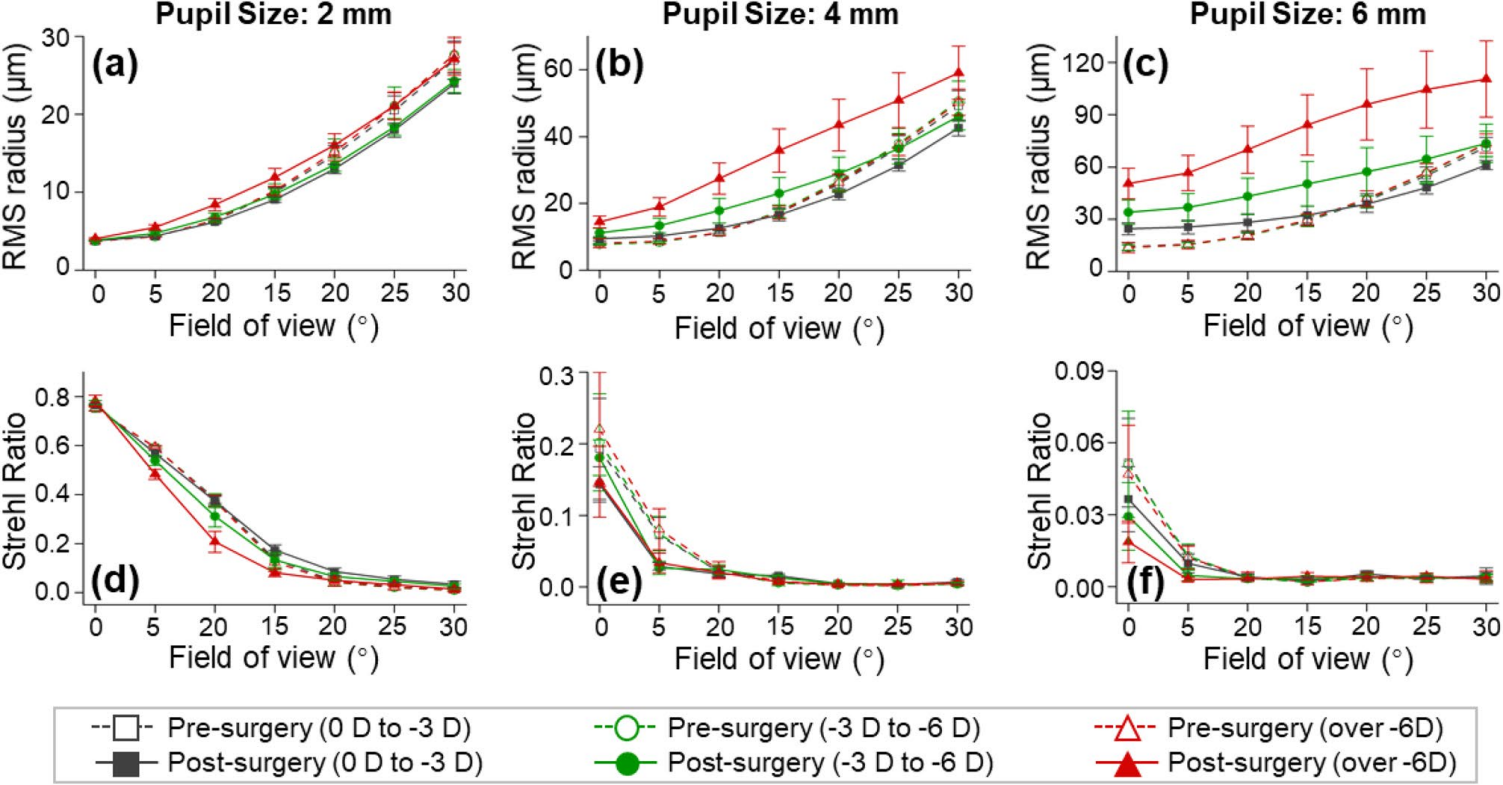
Spot size (**a**–**c**) and polychromatic PSF Strehl ratio (**d**–**f**) analysis (mean ± SD) at retina before and after femto-LASIK. The analysis was performed across the field of view (0°–30°), at 2-mm, 4-mm, and 6-mm pupil sizes, and for different initial myopic groups including mild myopia (0 to − 3 D), medium myopia (− 3 to − 6 D), and high myopia (over − 6 D), respectively.

The degree of spreading (blurring) of a point source at the image plane can be described by spot diagrams using the ray-tracing method and by polychromatic PSF using the FFT method. The Strehl ratio is defined as the ratio of the light at the peak of the diffraction pattern of an aberrated image to that at the peak of an ideal image. Figure 4a–c show the spot size evaluation results using the RMS radius values, and Fig. 4d–f evaluate the Strehl ratio for the polychromatic PSF. In general, the spot sizes were enlarged and the Strehl ratios were reduced as the field of view increased from 0° to 30°, indicating that the optical performance decreased from the central retina to the peripheral retina. When the pupil size was small (i.e., 2 mm), the optical performance was similar for all eyes before and after surgery. As the pupils enlarged from 4 to 6 mm, the pre-operative eyes had similar optical performance for all the myopic groups, but the post-operative eyes were significantly influenced by the initial myopia. After surgery, the optical performance of eyes that were initially more myopic tended to be much worse at larger pupil sizes, as demonstrated in Fig. 4c,d.

**Figure 5:**
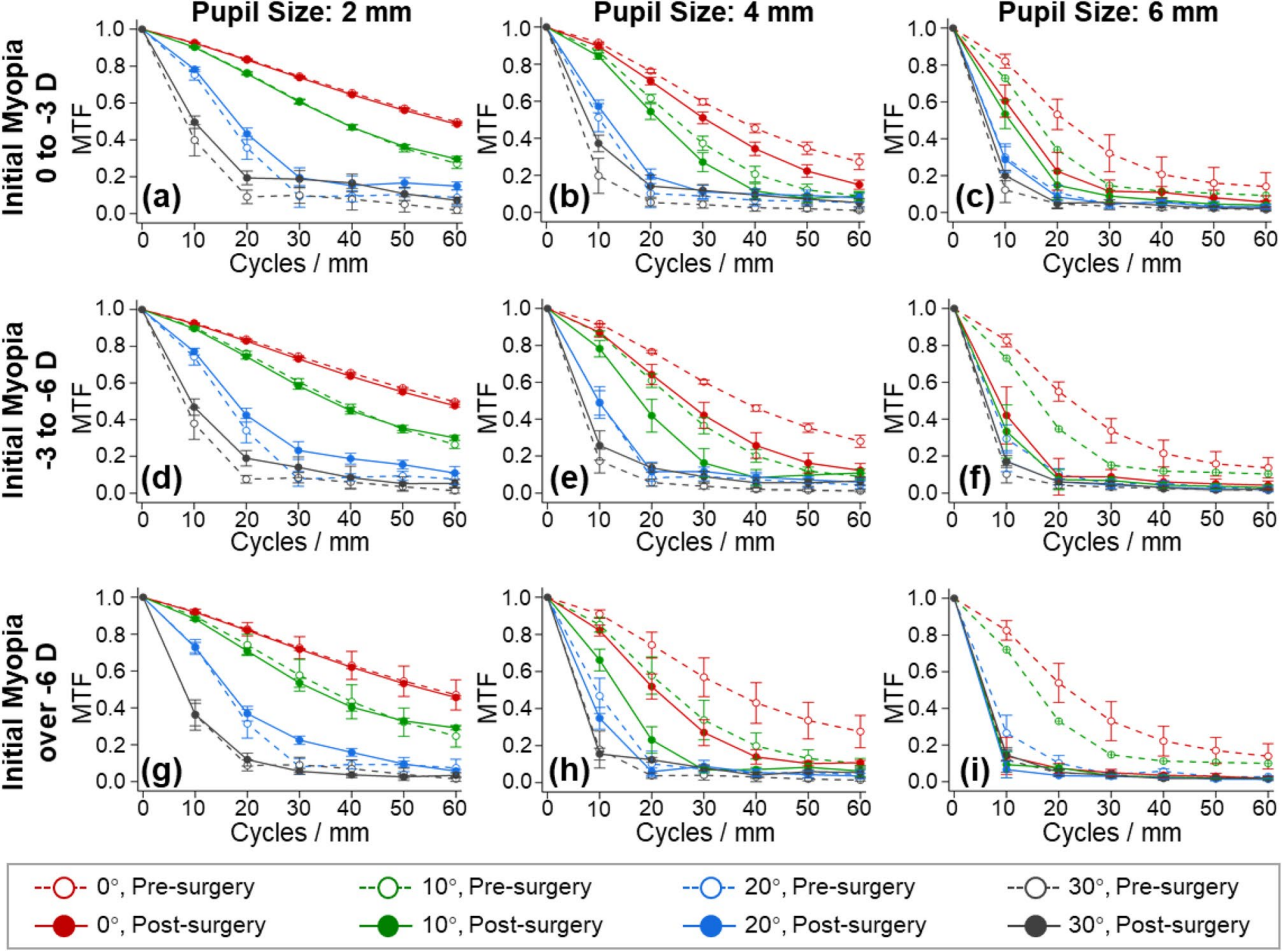
Polychromatic MTF analysis (mean ± SD) at the fields of 0°–30° at a maximum spatial frequency of 60 cycles per millimeter. The MTF values were averaged between the tangential and sagittal directions. (**a**–**i**) The MTF analysis at different pupil sizes (2 mm, 4 mm, and 6 mm) and for groups of different initial myopia, including mild myopia (0 to − 3 D), medium myopia (− 3 to − 6 D), and high myopia (over − 6 D).

Figure 5 presents the MTFs (mean values between the tangential and sagittal directions) for the pre- and post-surgical eyes at a maximum spatial frequency of 60 cycles per millimeter. In general, MTF declined as spatial frequency increased, and from central retina to peripheral retina. The post-surgical MTF was significantly affected by the initial myopia, and declined as the pupil size increased. MTFs of more myopic eyes tended to worsen at larger pupil sizes, as demonstrated in Fig. 5i. This result agreed with the spot size and PSF analysis (Fig. 4).

## Discussion

We have built customized eye models for 134 eyes before and after the femto-LASIK surgery, and we have provided quantitative and objective evaluations of changes in retinal imaging quality associated with the structural changes in the human eye induced by the surgery. The results of RMS spot size, Strehl ratio, and MTF analysis have shown that the optical performance is reduced from the central retina to the peripheral retina (e.g., from 0° to 30°). When the pupil size is small (e.g., 2 mm in diameter), the optical performance is similar for all eyes before and after surgery. As the pupil enlarges (e.g., up to 6 mm in diameter), the pre-operative eyes have similar optical performance for all the myopic groups, but the post-operative eyes are significantly influenced by the initial myopia. After femto-LASIK surgery, the optical performance with higher initial myopia tends to deteriorate at a larger pupil size. These results are consistent with previous studies on the influence of pupil size^14,49^ and initial myopia^50,51^ on the post-surgery optical performance. The post-operative chromatic aberrations also increase as the initial myopia increases after the surgery, but have different trends with the pupil size compared with other aberrations, such as monochromatic coma and spherical aberrations. The longitudinal chromatic aberrations after refractive surgery tend to decrease slightly as the pupil size increases, while the transverse chromatic aberrations remain similar for different pupil sizes. To the best of our knowledge, the finding of the post-surgical longitudinal chromatic aberrations decreasing with pupil size has not been presented previously. In general, when we consider the contributions to overall aberrations, pupil size and initial myopia are the two major factors that induce higher-order aberrations and decrease visual performance.

The accuracy of the eye model is dependent upon the precision of the measurements, especially the measurement of the crystalline lens. The human crystalline lens is a complex and inhomogeneous optical component with gradient refractive index distribution and aspheric surfaces^33^. We know that the lens shape and gradient index distribution may help to reduce the spherical aberration and coma that originate in the cornea^32^, but how the lens curvature and index gradient contribute to lens aberrations is still poorly understood due to a lack of reliable measurements on the lens shape outside of the central zone^37^. To date, the in vivo measurement of the posterior lens remains a longstanding challenge in the clinic. Optical methods, such as Scheimpflug photography^42^ and anterior-segment optical coherence tomography (OCT)^52,53^, are incapable of penetrating the iris tissue and imaging the peripheral region of the lens beneath the iris. Ultrasound^54^ and magnetic resonance imaging (MRI)^44^ can penetrate iris and sclera tissue but lack sufficient resolution to precisely depict the lens geometry. Previous studies have shown that the vertex radii of curvature of unaccommodated lens shapes are greater at the anterior surface than the posterior surface and decrease with age^55,56^. The radius and *Q*-values are age-dependent^25,42^ and are estimated in a wide range when the lens surfaces are fitted as conics. For example, the *Q*-values for the anterior and posterior surfaces of unaccommodated lenses were estimated as 2.46 and 1.09^43^, 4.27 and − 0.64^37^, − 3.13 and − 1^34^, 0 and − 3.25^29^, − 0.94 and 0.96^27^, and − 5.66 and 0.22^44^. The estimation differences of *Q*-values usually arose from the limited methods of measurement and the fitted diameter zone^37^. Simulation of the unaccommodated lens is already a great challenge, and enabling the eye model to account for the accommodation of the crystalline lens is far more difficult^34,44,56^. The measurement of retina shape is also limited by the optical distortions of the optical elements before the retina, particularly the gradient index distribution of the crystalline lens^57^. Therefore, better ocular measurement methods are required to improve the modeling accuracy of the human eye and to improve the simulation precision for the optical ray tracing method.

It should be noted that there is no perfect optical model of the eye that is best for every purpose^29^. Each of the previous eye models could be either a simple single-surface model to represent the on-axis chromatic and spherical aberration^26^ or a sophisticated model for anatomical accuracy^27^. An appropriate eye model is the one that gives valid results for a particular purpose, so a more complicated model is not necessarily better^29^. The main purpose of this study was to describe the effect of the post-surgical corneal structure change on visual performance. To meet this purpose, we prioritized the modeling of the anterior segment (especially cornea) while simplifying other parts of the eye, such as the lens, vitreous body, and retina. As is well known, the human eye is a decentered optical system with a non-rotationally symmetric structure^17^, where each element (e.g., cornea, pupil, and lens) can be decentered and tilted, and the photoreceptors (i.e., cones and rods) are not uniformly distributed at the retina^25,32,58^. We started the simulation of the human eye from a rotationally symmetric schematic eye model in an unaccommodated condition^29^. The asymmetricity of the anterior and posterior corneal surfaces can still be sufficiently represented by the surface heights in Zernike terms (e.g., tilt: Z_1_^±1^, astigmatism: Z_2_^±2^, and coma: Z_3_^±1^), but neither the tilt nor the decenter values of other ocular elements were presented in this model. We considered this as a valid simplification process. If we had used the empirically estimated (rather than trustworthily measured) decenter or tilt values for all the ocular elements of the model, it would have led to more system complexity and slower computing time, but it would not have improved the optical analysis accuracy. For the same reason, we assumed the gradient-index crystalline lens^25,27^ as homogeneous, and we assumed that all of the elements had homogeneous refractive indexes and Abbe-numbers^29^. Another issue is that we only evaluated the optical performance in each eye model, and ignored the neural factors that can also affect the visual performance. The combination of an individual’s optical and neural transfer functions could likely be better to predict the actual visual performance^23^. A neural transfer function expresses the loss of modulation as spatial frequency increases in the process of converting the optical signals to neural impulses and transferring the information through to the neural system and out as a percept^23^. Visual perceptual sensitivity is field-dependent due to the uneven distribution of photoreceptors and optic nerve cells from the fovea to the peripheral retina. This factor can be modeled optically using an apodization filter at the pupil plane^59^. Post-receptoral neural processing of the unevenly sampled retinal image affects the processing of blurred retinal images in a manner that can also be constructed as a form of apodization, or as a mathematical convolution of the optical PSF with a neural PSF^60^. The main focus of this study was the optical performance, but we will consider the effect of the interactive optical-neural performance on visual perception in our following studies. Although designed for Femto-LASIK, this eye modeling method could be utilized in other types of myopic surgeries^4^ and corneal surgeries, such as treatments of hyperopia^61^ and keratoconus^62^.

Corneal biomechanics, such as elasticity and viscosity, is another factor that may also contribute to the induction of high-order aberrations and the deterioration of optical performance. Integrating biomechanical factors into a more sophisticated eye model could potentially improve the accuracy of the refractive surgery prediction^63^. Corneal biomechanics maintain the ocular rigidity and are inherently tied to corneal health and visual performance^64^. Any morphological changes of the cornea are usually accompanied by biomechanical changes, and vice versa. Myopic refractive surgery may weaken the corneal strength and cause long-term instability^8,65^ and may rarely lead to several complications, such as corneal ectasia^66,67^; corneal biomechanics can, in turn, influence the predictability, stability, and safety of refractive surgery^68,69^. The pre-operative evaluation of corneal biomechanics is key to preventing ectasia as well as predicting and evaluating treatment outcomes^4^, but the clinical methods to precisely access corneal biomechanics are still limited. Developing more advanced imaging and quantitative methods, such as high-sensitivity optical coherence tomography (OCT)-based elastography methods, to more reliably access corneal biomechanics in clinic is an active research area in vision science and is of great interest to our research group^70–72^. Once the new corneal elastography tool and the measurable data of corneal biomechanics (such as natural frequency^73,74^ and Young’s modulus^75–77^) are provided, we could possibly provide an updated eye model that involves comprehensive information of ocular structure, biomechanics, optical performance, and neural functions. The application of such an eye model for refractive surgery could provide better pre-operative prediction of surgery outcomes, promote better surgical methods with improved visual performance, prevent post-surgical complications (e.g., corneal ectasia) more successfully, and reduce post-surgical patient complaints more effectively^78^.
